# Cryoablation of Extra-Abdominal Desmoid Tumors: A Single-Center Experience with Literature Review

**DOI:** 10.3390/diagnostics10080556

**Published:** 2020-08-04

**Authors:** Sarah Saltiel, Pierre E. Bize, Patrick Goetti, Nicolas Gallusser, Stéphane Cherix, Alban Denys, Fabio Becce, Georgia Tsoumakidou

**Affiliations:** 1 Department of Diagnostic and Interventional Radiology, Lausanne University Hospital and University of Lausanne, 1011 Lausanne, Switzerland; alban.denys@chuv.ch (A.D.); fabio.becce@chuv.ch (F.B.); georgia.tsoumakidou@chuv.ch (G.T.); 2 Department of Radiology, Clinique de Genolier, 1272 Genolier, Switzerland; pbize@genolier.net; 3 Department of Orthopaedics and Traumatology, Lausanne University Hospital and University of Lausanne, 1011 Lausanne, Switzerland; patrick.goetti@chuv.ch (P.G.); nicolas.gallusser@hopitalvs.ch (N.G.); stephane.cherix@chuv.ch (S.C.)

**Keywords:** aggressive fibromatosis, cryoablation, cryotherapy, extra-abdominal desmoid tumor, magnetic resonance imaging, treatment outcome

## Abstract

Cryoablation (CA) has gained popularity in the treatment of benign and malignant musculoskeletal tumors. While extra-abdominal desmoid (EAD) tumors are not malignant, they remain challenging to treat because of their high local recurrence rate. We reviewed all EAD tumors treated with CA at our institution between November 2012 and March 2020. Fourteen procedures were performed on nine females and one male (mean age, 33 ± 18 years) as either first-line (*n* = 4) or salvage therapy (*n* = 6) with curative intent (*n* = 8) or tumor debulking (*n* = 2). Mean tumor size was 63.6 cm^3^ (range, 3.4–169 cm^3^). Contrast-enhanced MRI was performed before treatment and at 3-, 6-, and 12-month follow-up. Treatment outcome was based on the change in enhanced tumor volume (ET-V). For curatively treated patients, the mean ET-V change was −97 ± 7%, −44 ± 143%, and +103 ± 312% at 3, 6, and 12 months, respectively. For debulking patients, the mean ET-V change was −98 ± 4%, +149 ± 364%, and +192 ± 353% at 3, 6, and 12 months, respectively. During a mean follow-up of 53.7 months (range, 12–83 months), one grade III and one grade IV complication were noted. We found CA to be safe and well tolerated in patients with EAD.

## 1. Introduction

Desmoid tumors (DTs) are rare, comprising <3% of all soft-tissue tumors. They are monoclinic, benign neoplasms that grow along musculoaponeurotic structures [1] and have no metastatic or malignant potential but are locally infiltrative and often recur after treatment [2,3]. Clinical manifestations are variable and highly unpredictable depending on the tumor location and speed of proliferation [4,5]. Most DTs arise sporadically from a b-catenin activation mutation in the *CTNNB1* gene [6], leading to decreased b-catenin protein degradation and its accumulation in the cytoplasm and nucleus of affected cells. Three discrete mutations have been identified (−41A, 45F, and 45P); the 45F mutation correlates with a higher risk of postoperative recurrence [7].

No consensus has been reached on the best treatment options. Surgical excision with wide margins is still considered the gold standard [8], but a recent paradigm shift towards other more conservative therapeutic approaches has occurred due to a high recurrence rate (up to 60%) and morbidity associated with surgery [9]. The 2019 global consensus guidelines from the Desmoid Tumor Working Group state that surgery should be avoided whenever possible and propose active surveillance as the front-line approach in cases of DT with no life-threatening or functional risk. DT can often regress spontaneously, with a spontaneous regression rate as high as 50% [1,10]. According to the 2019 guidelines, medical systemic, surgical, or radiotherapy treatments should be reserved in cases of tumor progression. The DT Working Group recommendations do not include thermal ablation therapies, though use of percutaneous cryoablation (CA) has increased in cases of extra-abdominal desmoid (EAD) tumors, with encouraging results [11,12,13,14,15]. The primary objective of the present study was to assess the safety and efficacy of CA for the treatment of EAD tumors using a percutaneous or combined ultrasound (US)-guided open surgery approach depending on the tumor location. As a secondary objective, we further reviewed the current literature on this subject.

## 2. Materials and Methods

### 2.1. Study Population and Design

After approval by the institutional ethics committee (Lausanne University Hospital CER-VD protocol 114/15, 8 February 2016), a retrospective search was performed in our regional sarcoma center database to identify cases from November 2012 to March 2020. Inclusion criteria were patients with histologically confirmed EAD tumors treated with CA and followed-up with MRI before and after treatment. Exclusion criteria were intra-abdominal DT or no available follow-up. Ten patients were identified (9 females and 1 male), with a mean age of 33 ± 18 years (range, 11–66 years) at the time of treatment. All patients had biopsy-proven DTs. One patient (patient no. 5) presented Gardner syndrome. Patients were referred for CA after multidisciplinary sarcoma tumor board discussion. No patient was excluded from the study. Patient demographics and DT localization/characteristics are shown in Table 1.

### 2.2. Treatment Procedures

Written informed consent for treatment was obtained from all patients before each procedure. All treatments were performed under general anesthesia by two experienced interventional radiologists using a percutaneous or combined US-guided open surgery approach. Whenever possible, complete ablation of the DT with 5–10 mm safety margins was attempted. A partial ablation was performed, as decided preoperatively, in cases of close proximity/direct contact of the lesion with vital anatomical structures (i.e., neurovascular bundles) or high risk of impaired functional status if complete ablation with safety margins is attempted. In such cases, CA was performed to relieve symptoms, halt tumor progression, or as a substitute for ongoing treatment (i.e., due to toxicity from systemic chemotherapy).

Strict surgical asepsis was used, and no antibiotic prophylaxis was administered. Three different 17-gauge CA probes (Ice-Seed, Ice-Sphere, and Ice-Rod) were used (SeedNet, Galil Medical Ltd., Yoqneam, Israel). Tumor length, size, and shape determined the number/type of CA probes and their configuration. A distance of 1–2 cm between probes was respected in order to achieve ice ball fusion.

#### 2.2.1. Percutaneous Cryoablation

Computed tomography (CT) with or without US guidance was used for tumor puncture. In all cases, direct puncture of the tumor was performed. In cases of DT proximity (distance < 1–2 cm) to neighboring organs at risk (i.e., nerves, vessels, or gastrointestinal and genitourinary tracts), supplemental thermal insulation/displacement techniques (hydrodissection, carbodissection) and/or nerve electrostimulation were used. Carbodissection and hydrodissection were performed by placing 21-gauge spinal needles between the tumor and the structure to be protected/displaced. Nerve electrostimulation was performed by placing an electrostimulation electrode in contact with the nerve at risk and proximal to the level of potential axonal damage.

**Figure 1:**
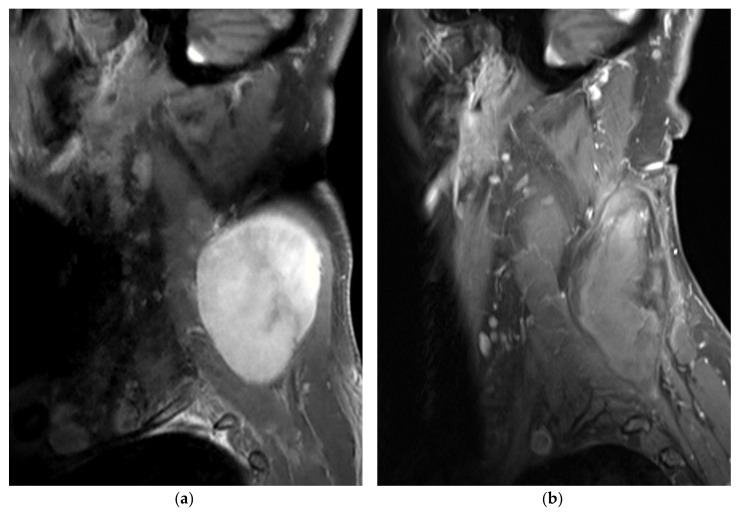

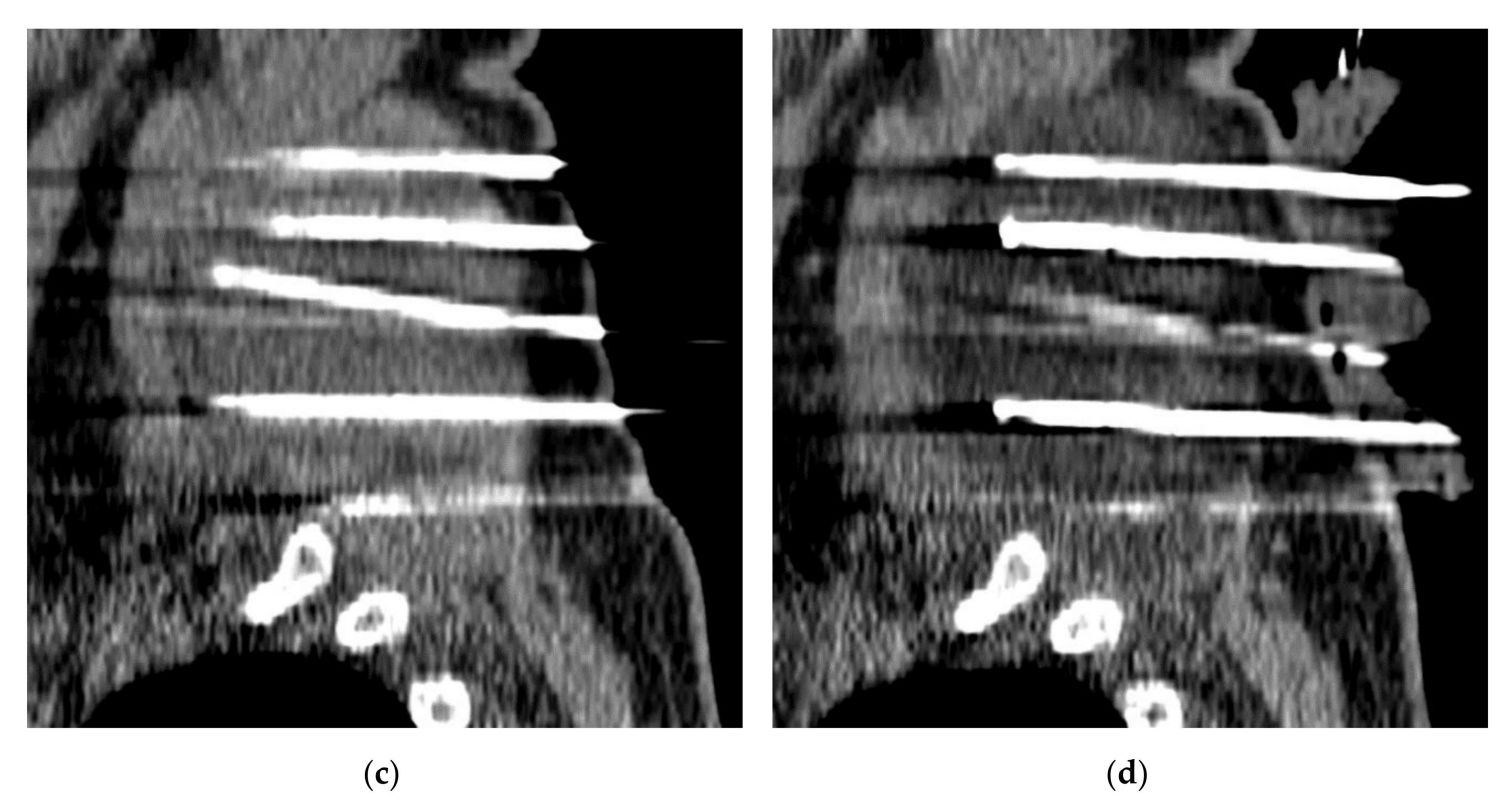
A 66-year-old male patient with extra-abdominal desmoid (EAD) tumor of the neck/cervical spine treated with percutaneous cryoablation (CA) (patient no. 4). (**a**,**b**) Sagittal gadolinium-enhanced fat-suppressed T1-weighted MR images prior to and 6 months after CA treatment, respectively. The desmoid tumor (DT) is well-delineated and homogeneously enhanced before CA. Six months after treatment, the DT has shrunk, and no residual tumor enhancement is seen; (**c**,**d**) Sagittal-reformatted CT images show several cryoprobes (19 in total) placed within the DT with the ice-ball (hypodense region) formation visible at the end of the second freezing cycle.

CA was generally performed using a double 10-min freeze protocol (two freezing cycles separated by a 9-min thawing cycle: 8-min passive + 1-min active). Intermittent CT controls during the freezing cycles were used to monitor ice ball extension (Figure 1). Duty cycle percentage (percentage on-off time, between 0 and 100%) and freezing cycle duration were adapted in cases of the ice ball approaching (<5 mm distance) a critical surrounding structure. Final CT images were obtained after cryoprobe retrieval to exclude any immediate complications, such as hematoma formation.

#### 2.2.2. Ultrasound-Guided Open Surgery Cryoablation

**Figure 2:**
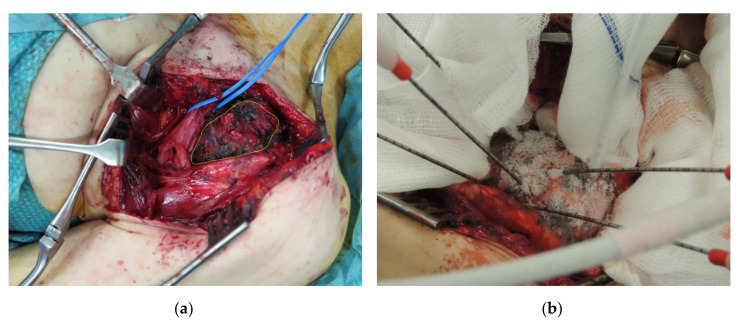
A 38-year-old female patient with EAD tumor of the left upper trunk/thoracic spine treated with open surgery CA (patient no. 7). (**a**) Intraoperative view of the DT (yellow outlines) with surgical exposition of the left brachial plexus (blue strings); (**b**) Positioning of six cryoprobes under US guidance with ice-ball formation and extension on the tumor surface.

In the surgical theater, the surgeon performed tissue dissection around the DT in order to achieve adequate exposure of the surgical field and surrounding neurovascular structures (Figure 2). The cryoprobes were then positioned inside the tumor under US guidance. The two freeze-cycle CA protocol was then applied. Ice ball monitoring was performed with both direct visualization and US. Whenever necessary, the surrounding tissues were warmed via direct warm saline instillation.

At the end of the procedure, patients from both treatment groups were transferred to the post-anesthesia care unit. Nonsteroidal anti-inflammatory drugs (NSAIDs) were systemically prescribed to control the usual post-CA inflammatory reaction.

### 2.3. Follow-Up

Clinical and imaging follow-up was performed before CA and 3, 6, and 12 months post-treatment and case by case thereafter. Routine clinical visits in electronic patient record files were reviewed retrospectively. Follow-up after the first year was more frequent in cases of residual tumoral lesions.

Imaging follow-up included contrast-enhanced magnetic resonance imaging (MRI) using our institution’s routine standardized soft-tissue tumor protocol: T1-weighted and T2-weighted axial images with fat-suppressed T1-weighted images in all three planes after gadolinium administration. MR images were reviewed retrospectively by one radiologist using a picture archiving and communication system workstation (Vue PACS, Carestream Health, Rochester, NY, USA). A residual tumor was defined as the presence or development of an enhancing nodule or crescent of tissue within the treatment zone. Any enhancement of the tumor was considered viable tumoral tissue. We considered a complete response (CR) to CA when there was no enhancement of the tumor within the treatment zone, partial response (PR) was a decrease in the enhanced tumor volume (ET-V) of ≥30% and progressive disease (PD) an increase of ≥20%, and ET-V change between −30% and +20% was considered stable disease (SD).

### 2.4. Data Collection and Analysis

The following data were collected: patient demographics (age, sex, and previous treatments; Table 1), tumor histology/genetic profile, tumor location/size, procedure-related parameters (type of anesthesia, number of cryoprobes/freezing cycles, and organs at risk/thermal insulation systems used), hospital stay duration, imaging, and clinical follow-up.

The total DT volume (TDT-V) and the ET-V, corresponding to a residual tumoral lesion, were measured using the dedicated PACS volume viewer tool at the different time intervals (before CA and 3, 6, and 12 months post-treatment). Using a free-hand method, the borders of the total DT and ET were first contoured on axial MR images (section thickness, 2–3 mm). The software then performed semi-automated 3D reconstructions in order to obtain the TDT-V and ET-V. We further determined CA success based on the absence or presence of any enhancing residual tumor at the 3-month follow-up: A0, no enhancing residual tumor; A1 and A2, enhancing residual tumor ≤15% and >15% of the initial TDT-V, respectively. For partial treatments, enhancing residual tumors were considered only if they were located within the ablation zone. Complications were reported using the Cardiovascular and Interventional Radiological Society of Europe (CIRSE) classification of adverse events [16].

### 2.5. Statistical Analysis

Descriptive statistics, including numbers and percentages, median and extreme values, or means and standard deviations where appropriate, were computed. Disease-free survival (DFS) was assessed using the Kaplan–Meier estimator (Statistics Kingdom, Melbourne, Australia).

## 3. Results

Ten patients underwent 14 CA procedures: 11 percutaneous and 3 using the combined US-guided open surgery approach. The mean TDT-V before treatment was 63.6 ± 58.5 cm^3^ (range, 3.4–169 cm^3^). In eight patients, complete ablation was attempted in either one (7 patients) or two (1 patient) consecutive sessions. In two patients, a partial ablation of the DT was attempted (decided preoperatively) because of the close proximity of the lesion to vital anatomical structures (1 patient) and the high risk of functional impairment or instability of the pelvis (1 patient). One patient from the curative group presented local recurrence near the radial nerve, which was further treated with partial ablation in a second session. Four out of 14 procedures involved treatment of a residual enhanced tumoral lesion discovered at the 3-month follow-up.

A mean of 7 cryoprobes were used per procedure (range, 3–19). Hydrodissection and carbodissection were applied in four procedures. Nerve electrostimulation was used in four procedures to assess nerve function when ablation was performed in close contact (<1 cm) to neighboring nerve roots. During one case with the combined approach, a satellite tumoral nodule was surgically excised with R0 surgical margins.

For curatively treated patients, the mean change in ET-V was −96.6 ± 6.8%, −44.1 ± 143.4%, and +103.4 ± 311.8% at the 3-, 6-, and 12-month follow-up, respectively (Table 2). For partially treated patients, the mean change in ET-V was −97.9 ± 3.6%, +148.6 ± 364.2%, and +192.1 ± 353.4% at the 3-, 6-, and 12-month follow-up, respectively (Table 2). The mean change in the TDT-V was +86.5 ± 106%, −16.3 ± 51.9%, and −28.8 ± 103.4% at the 3-, 6-, and 12-month follow-up, respectively, in the curative group, and +146.3 ± 229.5%, +113.2 ± 206.5%, +112.9 ± 208.6%, respectively, for partially treated patients.

In the curative group, 6 patients had CR and 2 PR at 3 months; 3 CR, 4 PR, and 1 PD at 6 months; and 3 CR, 1 PR, and 4 PD at 12 months. Regarding ablation success at 3 months, six of eight patients in the curative group had an A0 ablation, and the remaining two patients had an A1 ablation. For the group of patients with partial treatment, PD was noted in two cases, necessitating re-ablation (patients no. 7 and 10) and initiation of systemic therapy (patient no. 7). Regarding ablation success, we had one A0 and one A1 ablation. Patient no. 3 required systemic therapy to control PD 15 months after the second (partial) percutaneous CA treatment. Ablation success for the local recurrence in patient no. 3 was A0 at 3 months. Including the second treatment of patient no. 3, the partial treatment group had 2 cases of CR and 1 PR at 3 months; 1 CR, 1 PD, and 1 SD at 6 months; and 2 SD and 1 PD at 12 months.

At last follow-up, six patients had adjuvant chemotherapy (patients no. 3 and 5–9), 2 patients had surgical excision (patients no. 5 and 6), and one had an isolated limb perfusion. Mean follow-up was 53.7 ± 23 months (range, 12–83 months). One patient was lost to follow-up after 12 months.

**Figure 3:**
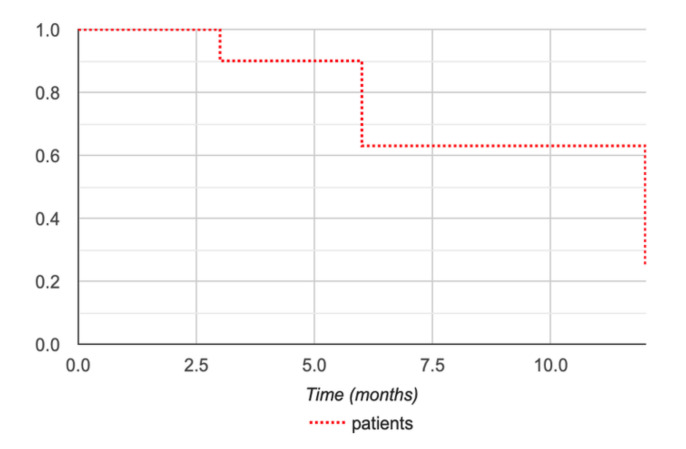
Kaplan–Meier disease-free survival (DFS) curve for EAD tumors after curative and partial CA treatments, up to 12-month MRI follow-up.

DFS was 90% at 3 months and 62% at 6 and 12 months for all patients (Figure 3).

Results on changes in pain level are reported in Table 3.

One grade III (colo-cutaneous fistula) and one grade IV complication (peroneal nerve palsy) were noted. The first necessitated clipping by a gastroenterologist 3 months after the CA procedure, with surgical excision of the fistula 5 months after the procedure because of persistent fistula. The second patient with the peroneal nerve palsy presented PD and was treated surgically with concomitant nerve grafting and tendon transfer 1.5 years after the initial CA procedure.

## 4. Discussion

No evidence-based or widely accepted guidelines on the management of EAD tumors are currently available. Surgical excision with wide margins has long been considered the gold standard [8], but due to its high recurrence rate (up to 60%) and associated morbidity, a paradigm shift towards other more conservative therapeutic approaches has occurred. Though no standard chemotherapy is available for DTs yet, promising new drugs (i.e., tyrosine kinase inhibitors, such as sorafenib and pazopanib) [17] and low-dose or conventional chemotherapeutic regimens (i.e., methotrexate plus vinblastine or vinorelbine and anthracycline-based regimens, respectively) [18,19] have been shown to shrink DTs. Nevertheless, the associated toxicity and unfavorable effects on quality of life are major drawbacks of the different chemotherapy regimens currently in use [20].

In recent years, percutaneous image-guided thermal ablation techniques (e.g., CA, radiofrequency, or microwave) have been valuable as minimally invasive curative and palliative therapies for benign and malignant tumors of different histologies and in various anatomical regions. The main advantage of CA over other thermal ablation techniques is that it allows clear visualization of the ablation zone (i.e., ice ball), certifying sufficient coverage of the targeted lesion while sparing the surrounding, non-targeted, cold-sensitive structures (e.g., colon, nerves, or ureters). CA utilizes cold thermal energy to freeze pathological tissue and achieve tumor destruction via both direct cellular and vascular injury/tissue ischemia [21]. Current CA systems use argon and helium gas passing through thin (14–17 gauge) probes to induce rapid freezing and thawing of tissue based on the Joule-Thompson effect. Gas expansion occurs in a small chamber inside the distal end of the cryoprobe. Argon provides a heat sink of approximately 9 kJ and can generate temperatures as low as −140 °C inside the ice ball, which expands by thermal conduction [22]. The surface of the visualized ice ball corresponds to the 0 °C isotherm, and the lethal isotherm rests a few millimeters inside of the ice ball boundary. Thus, a minimum 5 mm safety margin is needed to encompass the tumor in the −20 °C to −40 °C lethal isotherm. To further increase CA efficacy, freezing cycles should be repeated [23].

The recently published CRYODESMO-01 study is the first prospective, open-label, non-randomized, non-comparative, multi-center pilot study assessing CA in patients with progressive non-abdominal-pelvic DT [24]. The study met its primary endpoint with 86% of patients having non-progressive disease at the 12-month follow-up, reduced pain, and better functional status. We found an additional five retrospective case series including up to 34 patients, one prospective case series, and three case reports (Table 4) [11,12,13,14,24,25,26,27,28]. Most of these studies evaluated the safety and efficacy of CA as a second- or third-line treatment in symptomatic or progressive EAD tumors. In most cases, efficacy was evaluated using the RECIST criteria, TDT-V or ET-V measurements, and DFS rates. In up to 40% of cases, Bouhamama et al. reported that complete treatment was not possible due to the proximity of critical anatomical structures and the treatment’s main objective being tumor debulking [27]. Havez et al. reported that the DFS rate in a total of 13 patients (17 tumors) was stable at 82.3% at 6, 12, and 24 months [13]. Eight patients were treated partially with palliative goals and presented residual tumors at follow-up. Pain relief was initially reported in this patient group, but three patients presented progressive lesions, and two had recurrence of pain requiring anti-inflammatory therapy. In general, and although partial treatment can have a positive effect on pain/symptom palliation, most R1 excisions are associated with higher recurrence rates [19]. Schmitz et al. reported CR to percutaneous CA in 9 of 23 patients and residual tumors in the remaining 14 patients, with 4.3% PD after treatment [11].

Schmitz et al. demonstrated that CA can be safely repeated without increasing the complication rate [11]. Our patient group had four repeated procedures. Only one procedure performed in a patient with Gardner syndrome and multiple DTs on the abdominal wall was complicated by a colo-cutaneous fistula. Notably, carbodissection was used and the displacement of the bowel was considered sufficient during the freezing cycles. However, the patient presented abdominal pain and was diagnosed with fistula 3 months after the procedure.

Regarding the effect of CA on tumor volume, the literature data vary. Schmitz et al. found a reduced tumor volume in 22/23 patients of −2.7 to –100% over a follow-up of 16.2 months (range, 1.7 to 99.9 months) [11]. Kujak et al. reported some degree of reduction in tumor volume for 4/5 patients, but it was not significant [14]. Havez et al. treated 13 patients with 17 tumors in 17 percutaneous CA procedures, reporting a 73.5% reduction in tumor volume at the 6-month follow-up [13]. In our study, we observed a mean transient increase in TDT-V at 3 months on MRI that could be attributed to the post-treatment inflammatory reaction [29]. For the curative ablation group, a significant mean reduction in the ET-V was found at 3 and 6 months and a mean increase in the ET-V at 12 months. This increase was caused by PD in four patients. Subsequently, one patient was re-treated with partial ablation, two underwent surgical excision and adjuvant therapy, and two patients had adjuvant therapy. For the partial ablation group, we found a significant mean reduction in ET-V at 3 months and a mean increase in the ET-V at 6 and 12 months.

Kujak et al. stated that the appearance of EAD tumors on pretreatment MRI may be helpful in predicting the response to therapy [14]. The authors suggested that tumors with larger regions of high cellularity have greater potential for recurrence after surgical excision than those with greater proportions of fibrous tissue. Accordingly, we noted that the DTs in the two patients in the curative group with CR were hypointense on T2-weighted MR images, indicating DTs with low tumor cellularity [30].

CA of EAD tumors can be challenging due to the proximity of vital anatomical structures or their size; in most cases of DTs in the extremities, the lesion can be in direct contact with important nerves. Nevertheless, complication rates in the literature are low, ranging between 9.1% and 11.1% [31,32]. We faced two major complications, including that, in three cases, the distance between the DT and a major nerve was <1 cm. Havez et al. also reported peroneal nerve injury in one patient (5.8%) [13], and Schmitz et al. had three minor (Clavien-Dindo I) complications [11].

In a total of three patients with DT in direct contact with major neurovascular bundles, we used a combined US-guided open surgery CA approach as an alternative to the percutaneous approach to treat the tumors. We thought that exposing and protecting nerves could help avoid the development of iatrogenic complications related to freezing in such cases. No patient presented any adverse events. However, all three patients experienced tumor recurrence: one patient at 3 months and two patients at 6 months. Thus, it seems that an open surgery approach did not improve the oncological results, probably due to the lack of cross-sectional monitoring of the ice ball formation. In the future, we aim to treat similar DTs in dedicated interventional theaters combining open surgery with cross-sectional imaging.

The limitations of our study are related to the inherent bias of retrospective studies. In addition, we included only a small number of patients. Other limitations are the heterogeneity of the guidance modality (percutaneous versus open surgery) and the presence of concurrent additional treatments (e.g., chemotherapy or isolated limb perfusion). Therefore, the statistical analyses may be of limited value.

## 5. Conclusions

In this retrospective study, we found CA to be safe and well-tolerated in select patients with EAD tumors. In many cases, CA can be challenging because of the lesion size, but also because of the close proximity of critical anatomical structures. To reduce the risk of iatrogenic lesions, different thermal insulation/displacement techniques and neuromonitoring need to be applied. Combining open surgery with an image-guided approach can reduce adverse events, but further evaluation of the effect of this approach on treatment outcomes is needed. Prospective investigations with long-term follow-up are required to clarify the efficacy and safety profile of CA and further establish its place in the treatment of EAD tumors.

## Figures and Tables

**Table 1:** Patient demographics with clinical and treatment characteristics.

Patient No.	Age (Years)	Gender	Tumor Location	No. of CA Treatments	Type of CA Procedure (s)	Previous Treatment (s)	Follow-Up Time (Months)
1	19	F	Left buttock	1	Percutaneous	/	12
2	38	F	Right shoulder	1	Percutaneous	/	62
3	18	F	Left forearm	2	Open surgery + percutaneous	Surgery (R1) + chemotherapy	83
4	66	M	Neck/Cervical spine	1	Percutaneous	/	70
5	32	F	Inguinal folds	2	Percutaneous	Surgery	60
6	33	F	Right calf	1	Percutaneous	/	67
7	37	F	Trunk/Thoracic spine	2	Open surgery + percutaneous	Surgery + chemotherapy	67
8	17	F	Right thigh	1	Percutaneous	Chemotherapy	47
9	11	F	Left forearm	1	Open surgery	Chemotherapy	54
10	59	F	Left buttock	2	Percutaneous	Chemotherapy	16

CA = cryoablation; F = female; M = male.

**Table 2:** Outcome measures of total DT volume (TDT-V) and enhanced tumor volume (ET-V) changes after CA for curative and partial treatments.

CA Treatment Type	Patient No.	Volume Change (%) and Response Type	3 Months Follow-Up	6 Months Follow-Up	12 Months Follow-Up	Last Follow-Up
Curative	1	TDT-V	+105.7	−54.7	−63.4	−63.4
		ET-V	−100	−100	−100	−100
		Response	CR	CR	CR	CR
Curative	2	TDT-V	−8	−52.9	−57.5	−94.3
		ET-V	−82.2	−82	−81.6	−94.3
		Response	PR	PR	PR	PR
Curative	3	TDT-V	+14.3	+28.6	−5.6	/
		ET-V	−100	−71.4	+125	
		Response	CR	PR	PD	
Curative	4	TDT-V	+24.3	−18.9	−47.3	−77.5
		ET-V	−100	−100	−100	−100
		Response	CR	CR	CR	CR
Curative	5	TDT-V	+236.3	−46.9	−82.3	/
		ET-V	−100	−100	−100	
		Response	CR	CR	CR	
Curative	5 *	TDT-V	+299.5	+95.9	−92.3	+533.3
		ET-V	−100	−100	−100	
		Response	CR	CR	CR	PD (new lesions)
Curative	6	TDT-V	+74.8	−25.7	+12.6	−25.4
		ET-V	−87.4	+337.5	+793.8	−44.4
		Response	PR	PD	PD	PR
Curative	8	TDT-V	+65.5	−2.2	−25.7	−14.4
		ET-V	−100	−89.2	+73.3	−31.5
		Response	CR	PR	PD	PR
Curative	9	TDT-V	−33.3	−70	+66.7	−55.6
		ET-V	−100	−91.7	+420	−55.6
		Response	CR	PR	PD	PR
Partial	3 *	TDT-V	+76.5	−26.5	−2.9	−39.4
		ET-V	−100	−100	+0.8	−63.6
		Response	CR	CR	SD	PR
Partial	7 ^†^	TDT-V	−40.2	+15.5	−12.1	−93.3
		ET-V	−93.8	+566.7	+600	−92.2
		Response	PR	PD	PD	PR
Partial	10 ^‡^	TDT-V	+402.5	+350.4	+353.8	+353.8
		ET-V	−100	−21	−24.4	−24.4
		Response	CR	SD	SD	SD

* = second CA session for different tumoral lesions; ^†^ = second CA session for untreated tumoral part during initial CA procedure; ^‡^ = second CA session for tumoral recurrence.

**Table 3:** Patient-reported pain before and after CA.

Patient No.	Pain Before CA	Pain After CA
1	No	No
2	Yes	Partial relief
3	Yes	No
4	No	No
5	Yes	Yes, same
6	Yes	Yes, worse *
7	Yes	Partial relief
8	Yes	Yes, same
9	Yes	Yes, worse ^†^
10	Yes	Yes, same

* = fibular nerve injury during CA; † = progressive disease.

**Table 4:** Previous studies reporting EAD tumors treated with CA.

Reference	No. of Patients	No. of EAD Tumors	EAD Tumor Size Before CA	No. of CA Treatments	CA as First-Line Treatment	Mean Follow-Up Time (Months)	Imaging Follow-Up	Follow-Up Scheme (Months)	Partial Treatment with CA	Complete Treatment with CA	Residual Viable EAD Tumors	Reduction in EAD Tumor Volume at Last Follow-Up	No. of Recurrence/Disease Progression	Complications after CA	Symptoms before CA	Symptom Improvement after CA
[11]	18	26	1.6–118.3 cm^3^ (mean, 38.6 cm^3^)	31	2/26	16.2	MRI, CT	N/A	0	9/26	17/26	22/23	1/23	3/31	5/18	4/5
[12]	1	3	0.6–9.8 cm^3^ (mean, 6.5 cm^3^)	3	0	6	MRI	3, 6	0	3/3	0	3/3	0	0	1/1	1/1
[13]	13	17	1.7–14.2 cm (mean, 5.3 cm)	17	1/13	11.3	MRI	3, 6	8/17	9/17	8/17	16/17	2/17	3/13	N/A	14/17
[14]	5	5	3–10 cm (mean, 6.6 cm)	5	0	6–58	MRI, CT	N/A	2/5	2/5	3/5	4/5	1/5	N/A	5/5	3/5
[24]	50	N/A	0.6–1068 cm^3^ (mean, 111 cm^3^)	N/A	0	12	N/A	N/A	N/A	N/A	N/A	N/A	7/50	99.3%	N/A	83%
[25]	1	1	N/A	2	0	9	N/A	N/A	0	1/1	0	1/1	0	0	1/1	1/1
[26]	1	1	416 cm^3^	1	0	3	PET/CT	N/A	0	1/1	0	N/A	N/A	N/A	1/1	1/1
[27]	34	41	1–593 cm^3^ (mean, 104.4 cm^3^)													
[28]	23	23	0.4–456.5 cm^3^ (mean, 114.8 cm^3^)	30	14/23	15.4	MRI, CT	N/A	11/23	6/23	11/23	81% *	0/13 + 7/11	6/23	20/23	18/20
Current study	10	18	3.4–169 cm^3^ (mean, 63.6 cm^3^)	14	4/10	53.7	MRI	3, 6, 12	3/10	8/10	8/10	9/10	1/10	2/14	8/10	2/8

N/A = not applicable; * = average for 21 EAD tumors.
